# Editorial: Genetic Modification of Cardiac Tissue

**DOI:** 10.3389/fcvm.2019.00093

**Published:** 2019-07-16

**Authors:** Amadou K. S. Camara, David F. Stowe, Jin O-Uchi, Jason N. Bazil

**Affiliations:** ^1^Departments of Anesthesiology and Physiology, Medical College of Wisconsin, Milwaukee, WI, United States; ^2^Cardiovascular Division, Department of Medicine, Lillehei Heart Institute, University of Minnesota, Minneapolis, MN, United States; ^3^Department of Physiology, Michigan State University, East Lansing, MI, United States

**Keywords:** cardiovascular disease, gene editing, transgenic animal model, ischemia/reperfusion (I/R) injury, oxidative stress

Cardiovascular disease is associated with a myriad of molecular and genetic aberrations, but the details and impact of these changes on cellular and organ function are poorly understood (1). By utilizing recent advances in gene editing and analysis tools, we are entering an era of unprecedented discovery whereby the elucidation of the molecular underpinnings that link phenotype to genotype using animal models of cardiac disease is possible. This Research Topic entails original research articles that explore: (1) genetic and non-genetic contributions to cardiac abnormalities (Moumne et al.), (2) mechanism of mitochondrial centric cardioprotection involving a large conductance calcium activated potassium channel (BKCa) (Goswami et al.), (3) involvement of the mitochondria and endoplasmic reticulum crosstalk in the development of heart failure (Chen et al.), (4) a non-canonical role of telomerase in cardioprotection (Ait-Aissa et al.), and (5) the association of microRNA-1 with cardiac fibrosis (Valkov et al.). In addition to these excellent studies, two review articles on oxidant and antioxidant enzymes important in cardiovascular health and disease (Awad et al.) and technologies used to develop personalized models of heart failure for drug discovery are included (Greenberg et al.).

In the review by Awad et al., the importance of oxidant and antioxidant enzymes on cardiovascular health is discussed. In particular, they summarize our current understanding of major oxidant and antioxidant pathways and their roles in both heath and disease. The authors detail currently available animal models with knockout of genes encoding oxidant and antioxidant enzymes and include known effects and phenotypes. Lastly, they cover important limitations of using these animal models for translational research. Altogether, the review gives an excellent summary of what we know today about the oxidant and antioxidant enzymes associated with cardiovascular disease.

A review contribution by Greenberg et al. emphasizes the dire need for new and effective therapies for heart failure. They discuss the current challenges in heart failure research encompassing the heterogeneous nature of the disease, difficulty in obtaining tissue from heart failure patients, and the molecular changes that occur across time and space during disease progression. A promising solution to these problems is the culturing of human pluripotent stem cells. This method retains the genetic background and allows for the development of patient specific therapies. However, cells in culture are quite distinct and behave differently from those imbedded in native tissue matrices. To circumvent this problem, the authors go into exquisite detail describing the state-of-the-art advances using 3D human engineered heart tissue as screening and rapid development platforms for drug discovery. The development of these platforms could greatly advance our understanding of the causes and development of heart failure and provide impetus to carry research forward from bench to bedside.

The study by Moumne et al. provide compelling evidence that gestational hypoxia is strongly linked to cardiac malformations. By breeding wild-type females with heterozygous Nkx2-5 mutant knock-in mice (Nkx2-5^+^/^R52G^) (2) in males at normoxic and hypoxic conditions, they were able to show similar cardiac deformities between the hypoxic control and heterozygous animals. The transcription factor Nkx2-5 is critical for normal heart formation and development. Mutations in this gene are known to cause atrial septal defects, tetralogy of Fallot, and hypoplastic left heart syndrome. This study demonstrates that non-genetic and genetic cardiac anomalies may share a common mechanism related to Nkx2-5 function. If true, this novel observation will open new therapeutic avenues for patients with congenital heart defects.

Goswami et al. conducted a study on cardioprotection that explores the role of a large conductance, calcium activated potassium channel (BKCa) expressed in mitochondria. They employed an *ex vivo* Langendorff model of ischemia/reperfusion (I/R) injury using wild-type mice and mice overexpressing a gain-of-function subunit of BKCa (BKR207Q) (3). Genetic activation of the BKCa did not alter cardiac hemodynamics, but it reduced free radical generation after I/R stress. While pharmacological data has provided evidence for BKCa's role in cardioprotection, this study definitively demonstrates that this ion channel located in the inner mitochondrial membrane is a major component of the endogenous cardioprotection pathway.

Using a cardiac specific knockout of the tumor suppressor gene p53, Chen et al. revealed a novel cardioprotective pathway. They generated cardiac specific p53 KO mice by crossing floxed p53 mice with mice carrying α-myosin heavy chain *Cre*. After a 48-h thapsigargin treatment to induce ER stress, cardiomyocyte death in p53 KO mice decreased relative to wild-type mice. In addition, cardiac mitochondria from p53 KO mice retained complex I activity and pyruvate dehydrogenase content. Furthermore, the release of cytochrome c and apoptosis inducing factor was also inhibited in cardiac mitochondria from p53 KO mice after thapsigargin treatment. These findings provide an additional target to the arsenal of treatments for cardiac injury and heart failure.

A fresh insight into cardioprotective mechanisms was provided by Ait-Aissa et al. on the importance of a telomere-independent function of telomerase in cardiac I/R injury. Using CRISPR/Cas9 technology, they generated transgenic rats with the catalytic-subunit of telomerase knockout (TERT^−/−^). In an *ex vivo* Langendorff experimental I/R model, they showed that TERT^−/−^ paradoxically reduced myocardial infarction, but their mitochondrial function was impaired and they showed increased mitochondrial lesion when compared to wild-type. In addition, TERT^−/−^ rats were more susceptible to I/R injury compared to the wild-type when both rat groups were treated with angiotensin II. In the presence of angiotensin-II infusion, infarct sizes increased after I/R and were even larger in the TERT^−/−^ rats compared to the wild-type. This study opens up a new area of investigation regarding the mechanism behind the protective effect of TERT on cardiac tissue exposed to stressors such as I/R injury and in chronic heart disease.

While miRNA-1 has long been established as a muscle specific miRNA, Valkov et al. identified a new role of this transcript in cardiac fibroblasts. Using chronic (4 weeks) subcutaneous injection of angiotensin II, they demonstrated a significant downregulation of miRNA-1 in cardiac fibroblasts and an increase in cardiac fibrosis in Sprague-Dawley rats. They validated this finding by overexpressing miRNA-1 level using adenovirus encoding miRNA-1 or by reducing miRNA-1 level using chemically modified sequence-specific miRNA-1 antagomir in primary fibroblasts *in situ* to inhibit or facilitate fibroblast proliferation, respectively. They further identified cyclin D2 and cyclin-dependent kinase 6 as the downstream targets of this transcript. From this study, there is evidence of an additional regulatory function of miRNA-1 in the heart that may furnish a new strategy to treat cardiac fibrosis in patients with heart failure.

In summary, with the advent of gene editing tools and other molecular approaches, our understanding of cardiovascular physiology has immensely grown to the point where we are able to now design effective therapeutic strategies that hold promise in minimizing the effects of heart disease. Overall, the work in this Research Topic has contributed to our understanding of some of the underlying mechanisms that are the basis of cardiovascular disease by utilizing a range of novel genetic and molecular tools. We are now in an exciting time with new technological developments in drug design and therapeutics for enhanced cardiovascular health (4).

## Author Contributions

JB, AC, DS, and JO-U contributed conception and design of this topic. All authors contributed to revision, read, and approved the submitted version.

### Conflict of Interest Statement

The authors declare that the research was conducted in the absence of any commercial or financial relationships that could be construed as a potential conflict of interest.
